# Integrating Immune Checkpoint Inhibitors With Total Neoadjuvant Therapy in Proficient Mismatch Repair Rectal Cancer

**DOI:** 10.1002/ags3.70083

**Published:** 2025-08-24

**Authors:** Yoshinori Kagawa, Jun Watanabe, Koji Ando, Caleah Kitchens, Aron Bercz, J. Joshua Smith

**Affiliations:** ^1^ Department of Gastroenterological Surgery Osaka International Cancer Institute Osaka Japan; ^2^ Department of Colorectal Surgery Kansai Medical University Osaka Japan; ^3^ Department of Surgery and Science Kyushu University Fukuoka Japan; ^4^ Department of Surgery, Colorectal Service Memorial Sloan Kettering Cancer Center New York New York USA

**Keywords:** immune checkpoint inhibitors, locally advanced rectal cancer, microsatellite stable tumors, proficient mismatch repair, total neoadjuvant therapy

## Abstract

The management of locally advanced rectal cancer (LARC) has evolved with the adoption of total neoadjuvant therapy (TNT), integrated chemoradiotherapy (CRT) or short‐course radiotherapy (SCRT) with systemic chemotherapy. Although immune checkpoint inhibitors (ICIs) show remarkable efficacy in mismatch repair‐deficient/MSI‐H colorectal cancer, their role in proficient mismatch repair (pMMR)/microsatellite stable (MSS) tumors remains limited owing to poor immunogenicity. CRT or SCRT has emerged as a promising immunomodulator capable of converting “cold” pMMR/MSS tumors into “hot” immune‐responsive environments, thereby enhancing antigen presentation and PD‐L1 expression. Although CRT‐ICI combinations have achieved modest efficacy with pathological complete response (pCR) rates generally plateauing around 40%, recent studies that incorporate ICIs into TNT (TNT‐ICI), notably UNION, TORCH, and PRECAM, have achieved higher pCR and clinical complete response (cCR) rates (40%–60%). Focusing exclusively on TNT, this review underscores that optimal sequencing and chemotherapy intensity are paramount for maximizing synergy while limiting lymphodepletion. It consolidates the growing clinical evidence and mechanistic rationale for integrating ICIs into TNT, for pMMR/MSS LARC, and delineates a pathway toward higher pCR and cCR rates alongside organ‐preserving treatment strategies.

## Graphical abstract

The key to unlocking ICI benefit in pMMR/MSS LARC is to convert “cold” tumors into “hot” ones through lymphocyte‐sparing SCRT and judicious chemotherapy, creating a milieu in which ICIs can act effectively.

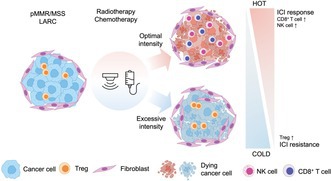

## 1 Introduction

The management of locally advanced rectal cancer (LARC) has markedly transformed with the adoption of total neoadjuvant therapy (TNT) [1]. This strategy—integrating neoadjuvant chemoradiotherapy (CRT) or short‐course radiotherapy (SCRT) with systemic chemotherapy prior to surgery—enhances tumor regression, disease control, and organ preservation. Trials including RAPIDO [2], STELLAR [3], PRODIGE‐23 [4], and OPRA [5] have provided key insights into optimizing TNT for LARC.

Immunotherapy targeting programmed cell death protein 1 (PD‐1) and its ligand PD‐L1 has demonstrated marked efficacy in mismatch repair‐deficient (dMMR) and MSI‐H colorectal cancers [6]. A study on dostarlimab in dMMR/MSI‐H rectal cancer reported a 100% complete clinical response (cCR), preventing surgery in all patients [7]. These findings highlight the potential of immunotherapy‐driven organ‐preserving strategies. However, most rectal cancers are proficient mismatch repair (pMMR) or microsatellite stable (MSS) and respond poorly to immune checkpoint inhibitors (ICIs) [8].

Combination with CRT or SCRT and ICIs has been investigated as a potential therapeutic strategy. Radiotherapy can modulate the immune environment, enhancing antigen presentation, T‐cell infiltration, and PD‐L1 expression, transforming “cold” tumors into “hot” tumors [9, 10]. These effects have spurred interest in pairing radiotherapy with ICIs to overcome resistance in pMMR/MSS rectal cancers. Nevertheless, despite these immunologic advantages, reported pathologic complete response (pCR) rates with CRT or SCRT plus an ICI alone (without systemic chemotherapy) plateau at roughly 40%, underscoring the need for further optimization [11].

Therapeutic focus has shifted from pairing ICIs with standalone SCRT or CRT to integrating ICIs into TNT. In the phase III UNION trial, a TNT regimen that comprised SCRT followed by CAPOX and camrelizumab achieved a 39.8% pathologic complete‐response rate, compared with 15.3% for CRT plus camrelizumab, reinforcing the move toward TNT‐based ICI strategies [12].

This mini review focuses only on TNT plus ICI (TNT‐ICI) in pMMR/MSS rectal cancer, not CRT‐ICI; it delivers the first up‐to‐date synthesis after UNION, covering SCRT based‐TNT versus CRT‐based TNT, sequencing, and non‐operative management (NOM).

## 2 Rationale for ICIs in pMMR/MSS LARC

The introduction of ICIs targeting PD‐1 and its ligand, PD‐L1, has transformed the management of various malignancies, particularly dMMR/MSI‐H colorectal cancer [13]. Patients with dMMR tumors exhibit high tumor mutational burden (TMB), increased neoantigen presentation, and a preexisting inflammatory tumor microenvironment, making them highly responsive to ICIs. Clinical trials of dMMR/MSI‐H colorectal cancer [14, 15] have demonstrated remarkably high complete response (CR) rates, prompting the regulatory approval of ICIs for this subgroup. However, dMMR/MSI‐H tumors constitute only a minority of rectal cancer cases, with the majority being pMMR/MSS tumors, which exhibit limited responsiveness to ICIs.

The poor efficacy of ICI in pMMR/MSS rectal cancer is largely attributed to the immunologically “cold” nature of these tumors [16]. Unlike dMMR/MSI‐H tumors, pMMR/MSS tumors typically exhibit low TMB, reduced neoantigen load, and minimal cytotoxic T cell infiltration. These tumors often exhibit an immunosuppressive tumor microenvironment (TME) characterized by a high density of T regulatory cells (Tregs), tumor‐associated macrophages, and myeloid‐derived suppressor cells (MDSCs), which inhibit T cell activation and promote immune evasion [17]. Additionally, limited PD‐L1 expression in most pMMR/MSS tumors restricts the effectiveness of the PD‐1/PD‐L1 blockade alone.

Consequently, the attention has shifted to combination strategies to enhance the immunogenicity of pMMR/MSS tumors and modulate the TME to improve ICI responsiveness [9], and integrating radiotherapy with ICIs is a promising approach. Radiotherapy induces immunogenic cell death, increases tumor antigen presentation, and promotes T‐cell infiltration into tumors (tumor‐infiltrating lymphocytes [TILs]). Additionally, it upregulates PD‐L1 expression and activates the stimulator of the interferon gene pathway, enhancing dendritic cell activation and facilitating tumor antigen cross‐presentation [18].

Hypofractionated SCRT has emerged as an especially attractive platform for ICI integration because its brief schedule limits treatment‐induced lymphopenia [11]. In pre‐clinical models, high‐dose hypofractionated irradiation (8 Gy × 2) preserved intratumoral and peripheral CD8^+^ T‐cell infiltration and activation while simultaneously depleting granulocytic myeloid‐derived suppressor cells. It also up‐regulated interferon‐stimulated genes—including MHC class I and PD‐L1—thereby priming the tumor for checkpoint blockade; when combined with anti‐PD‐1 therapy, this regimen reversed adaptive immune resistance and produced superior tumor control [19]. A recent clinical meta‐analysis corroborates these findings: SCRT combined with ICIs achieved a 51% pCR rate versus 30% for conventional CRT plus ICIs, suggesting that hypofractionation may synergize more effectively with immunotherapy in pMMR/MSS disease [11]. Prospective studies further show that hypofractionated schedules preserve absolute lymphocyte counts by ~30% relative to 45–50 Gy CRT, supporting SCRT as a lymphocyte‐sparing platform for immuno‐oncology [20]. Compared with CRT, SCRT therefore causes less prolonged lymphocyte depletion and allows more rapid recovery of effector cells required for PD‐1/PD‐L1 blockade [20]. Maintaining systemic immunity is critical for maximizing radiotherapy–ICI synergy and for generating durable antitumor responses [21].

**FIGURE 1:**
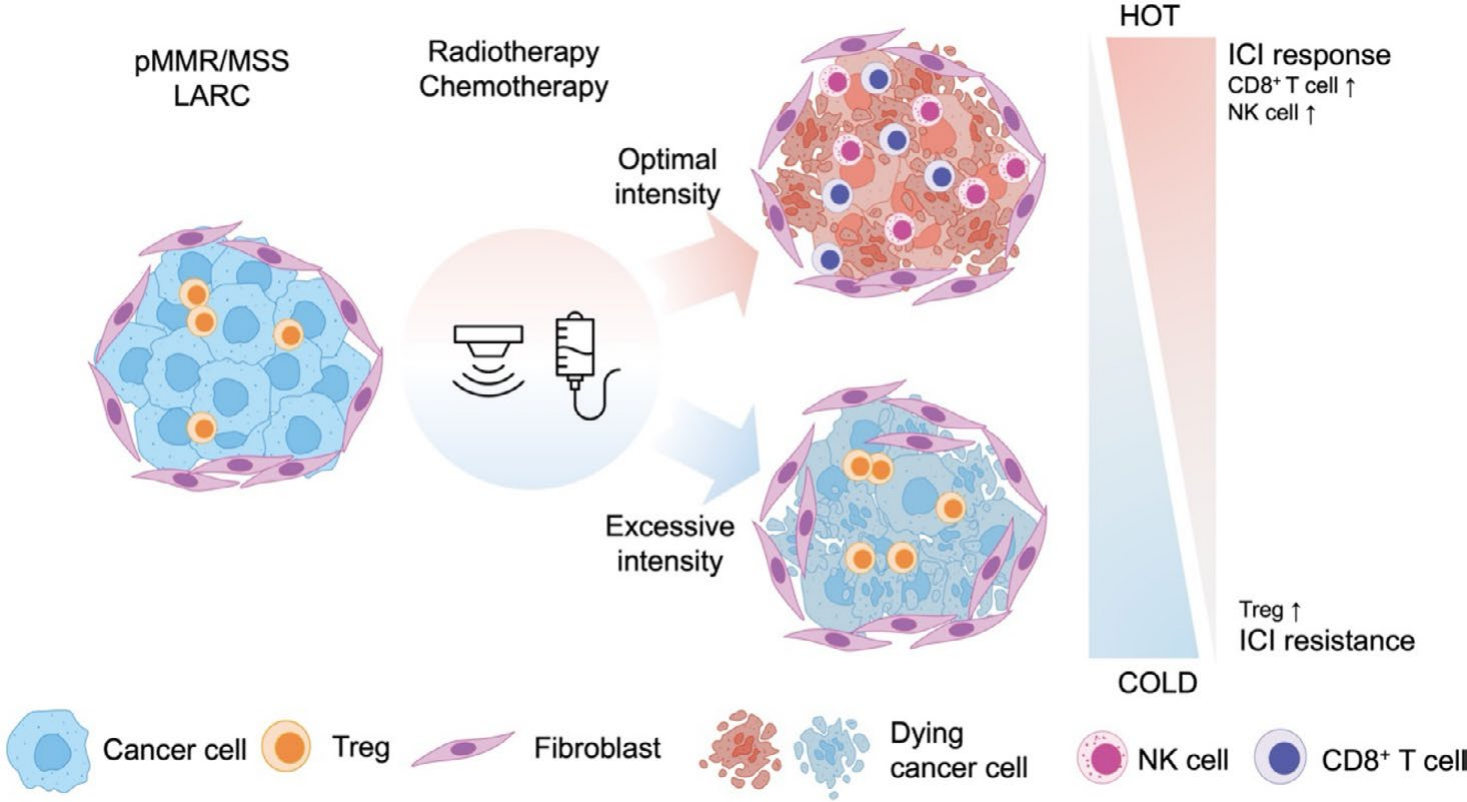
Radiotherapy and chemotherapy exert both immunostimulatory and immunosuppressive effects, modulating the balance between immune activation and suppression within the tumor microenvironment. Radiotherapy and chemotherapy enhance anti‐tumor immunity by inducing immunogenic cell death (ICD), releasing damage‐associated molecular patterns (DAMPs). These molecules activate dendritic cells, which present tumor antigens to CD8^+^ T‐cells, triggering a potent immune response. The release of inflammatory cytokines, such as IFN‐γ, further promotes the recruitment and activation of CD8^+^ T‐cells and natural killer (NK) cells, strengthening the overall immune response. Radiotherapy can induce a systemic abscopal effect, where localized radiation stimulates immune responses against distant tumor sites, particularly when combined with immune checkpoint inhibitors (ICIs). However, depending on treatment intensity, radiotherapy and chemotherapy can also contribute to immune suppression. High‐dose radiation therapy promotes secretion of immunosuppressive cytokines such as TGF‐β and IL‐10, expanding Tregs and counteracting CD8^+^/NK responses—particularly when total dose or field size is excessive. Moreover, lymphodepletion, a consequence of excessive radiotherapy and chemotherapy, reduces circulating lymphocytes and weakens systemic anti‐tumor immunity, diminishing the effectiveness of immunotherapy.

Chemotherapy contributes significantly to this combination strategy by inducing immunogenic cell death and reducing immunosuppressive cell populations such as Tregs and MDSCs [22, 23]. However, over‐intensive chemotherapy may paradoxically impair anti‐tumor immune responses [24]. Chemotherapy‐induced lymphodepletion can diminish ICI efficacy by reducing T cell populations critical for immune activation. Excessive exposure to radiation and chemotherapy may result in T cell depletion and fibrosis, counteracting the immunostimulatory effects of radiotherapy. Therefore, optimizing treatment intensity and sequencing is essential to maximize the benefits of ICIs in pMMR/MSS LARC (Figure 1).

## 3 Clinical Evidence Supporting TNT–ICI Integration

Recent clinical evidence indicates that incorporating ICIs into TNT can meaningfully enhance short‐term outcomes in pMMR/MSS rectal cancer and may widen opportunities for NOM (Table 1). In CRT–based TNT, a randomized phase II trials showed that adding a PD‐1 inhibitor almost doubled the CR rate: sintilimab combined with CAPOX‐CRT increased the composite pCR or cCR to 44.8% from 26.9% [25]. Single‐arm studies such as NECTAR (tislelizumab) corroborate the activity of CRT plus ICIs [26], although the pembrolizumab arm of NRG‐GI002 reported higher grade 3–4 toxicities than the control arm [27].

**TABLE 1:** Current clinical evidence on integrating immune checkpoint inhibitors with total neoadjuvant therapy.

Trial name	NCT number	Phase	Registration year	Study population and size	Study design	Treatment plan/intervention	Primary endpoint	Results
Neoadjuvant chemoradiotherapy with or without PD‐1 antibody sintilimab	NCT04304209	II	2020	Clinical stage II–III rectal cancer; *N* = 134	Randomized	Arm A: (CAPOX + sintilimab) × 4 + LCRT (50 Gy/25 F) Arm B: (CAPOX ×4) + LCRT (50 Gy/25 F)	CR Rate (pCR + cCR)	Arm A, CR rate: 44.8% (95% CI: 32.6%–57.0%) Arm B, CR rate: 26.9% (95% CI: 16.0%–37.8%) Response Ratio: 1.667 (95% CI: 1.035–2.683)
UNION	NCT04928807	III	2021	T3‐4/*N*+ rectal cancer; *N* = 231	Randomized	Arm A: SCRT (25 Gy/5 F) + (CAPOX + camrelizumab) × 2 Arm B: SCRT (25 Gy/5 F) + (CAPOX ×2)	pCR Rate	Arm A, pCR rate: 39.8% (95% CI: 30.7%–49.5%) Arm B, pCR rate: 15.3% (95% CI: 9.3%–23.0%) Odds Ratio: 3.7 (95% CI: 2.0–6.9)
TORCH	NCT04518280	II	2020	T3‐4/*N*+ rectal cancer; *N* = 130	Randomized	Arm A: SCRT (25 Gy/5 F) + (CAPOX + toripalimab) × 6 Arm B: (CAPOX + toripalimab) × 2 + SCRT (25 Gy/5 F) + (CAPOX + toripalimab) × 4	CR Rate (pCR + cCR)	Arm A, CR rate: 56.5% Arm B, CR rate: 54.2%
PRECAM	NCT05216653	II	2022	Clinical stage II–III rectal cancer; *N* = 34	Single‐arm	SCRT (25 Gy/5 F) + (CAPOX ×2 + envafolimab ×6)	pCR Rate	pCR rate: 62.5% (20/32)
NECTAR	NCT05245474	II	2022	Clinical stage II–III rectal cancer; *N* = 186	Single‐arm	LCRT (50 Gy/25 F) with capecitabine ×3 with tislelizumab ×3	pCR Rate	pCR rate: 40.0% (95% CI: 27.61%–53.82%)
Averectal	NCT03503630	II	2018	Clinical stage II–III rectal cancer; *N* = 40	Single‐arm	SCRT (25 Gy/5 F) + (FOLFOX ×6 + avelumab)	pCR Rate	pCR rate: 37.5% (15/40)
NRG‐GI002	NCT02921256	II	2016	Clinical stage II–III, distal (< 5 cm from anal verge) rectal cancer; *N* = 185	Randomized	Arm A: FOLFOX ×6 + LCRT (50.4 Gy with capecitabine) Arm B: FOLFOX ×6 + LCRT (50.4 Gy with capecitabine and pembrolizumab)	NAR Score	Arm A vs. Arm B Mean NAR score: 14.08 (95% CI: 10.74–17.43) vs. 11.53 (95% CI: 8.54–14.51); *p* = 0.26 pCR rate: 29.4% vs. 31.9%; *p* = 0.75 cCR rate: 13.6% vs. 13.9%; *p* = 0.95

*Note:* This table lists representative studies identified through a search of ClinicalTrials.gov conducted on 20 April 2025.

Abbreviations: CAPOX: capecitabine + oxaliplatin; cCR, clinical complete response; CI, confidence interval; FOLFOX, fluorouracil + leucovorin + oxaliplatin; LCRT, long‐course chemoradiotherapy; NAR, neoadjuvant rectal cancer; pCR, pathological complete response; SCRT, short‐course radiotherapy.

SCRT appears even more immunogenic in the TNT setting. The phase III UNION trial demonstrated superior pCR and cCR rates—39.8% and 41.6%, respectively—when SCRT was followed by camrelizumab and CAPOX, compared with 15.3% and 18.6% with CRT plus CAPOX [12]. In TORCH, toripalimab delivered after SCRT based TNT achieved a 50% pathological CR and a 43.5% cCR, whereas starting the antibody before SCRT reduced the cCR to 35.6%, underscoring the importance of sequencing [28]. PRECAM, which combined SCRT with abbreviated CAPOX and prolonged envafolimab, yielded a notable 62.5% pCR [29], while Averectal (SCRT, FOLFOX and avelumab) produced a 37.5% pathological CR [30].

Most TNT protocols administer ICIs after radiotherapy in a consolidation phase, a strategy supported by pre‐clinical data showing that irradiation induces immunogenic cell death, up‐regulates PD‐L1 and expands neo‐antigen display, thereby priming tumors for checkpoint inhibition. Clinical trials that start ICIs within days to weeks of completing SCRT, such as UNION [12] and Averectal [30], have documented rapid tumor‐infiltrating‐lymphocyte influx and encouraging response rates. In contrast, induction or concurrent ICI schedules remain investigational, with inconsistent results observed in studies like NECTAR [26] and NRG‐GI002 [27], underscoring the need for prospective immune monitoring to determine the optimal timing.

Two themes emerge. First, across contemporary pMMR/MSS trials, SCRT‐based TNT with ICIs tends to achieve higher CR rates (approximately 40%–60%) than CRT‐based TNT with ICIs, likely because SCRT minimizes lymphopenia while enhancing PD‐L1 expression. Second, the precise timing of ICI administration relative to SCRT and chemotherapy can alter CR rates by more than ten percentage points, directly influencing eligibility for NOM strategies. Although these insights identify sequencing and fractionation as key levers for further improvement, long‐term survival data from ongoing phase III trials remain essential before TNT plus ICIs can be embraced as a definitive standard of care.

## 4 Ongoing TNT–ICI Trials for pMMR/MSS LARC

Multiple trials are currently evaluating ICI–TNT combination in patients with pMMR/MSS LARC to optimize treatment sequencing, improve response, and support nonoperative strategies (Table 2). STELLAR II (NCT05484024) is a phase III trial (*N* = 588) comparing SCRT + CAPOX/FOLFOX with or without sintilimab to investigate whether ICIs enhance CR rates, allowing NOM. PRECAM‐R (NCT05752136) is a phase III trial (*N* = 108) comparing SCRT + CAPOX with or without envafolimab to assess pCR as the primary outcome and evaluate de‐escalation strategies. TNTi (NCT06229041) is a phase III study (*N* = 472) testing TNT with or without camrelizumab in high‐risk patients with LARC. The primary endpoint of this study is pCR, focusing on PD‐1 blockade in aggressive diseases. UNICORN (NCT05922587) is a phase III trial (*N* = 280) comparing SCRT + camrelizumab and CAPOX with standard LCRT, evaluating oncological and functional outcomes. TORCH‐2 (NCT06166016) is a phase III study (*N* = 220) examining SCRT followed by toripalimab and chemotherapy in various sequences, integrating PD‐L1/TILs as biomarkers. NECTAR‐2 (NCT06235599) is a phase II study (*N* = 50) using tissuelizumab + oxaliplatin followed by chemoradiotherapy to assess pCR and the role of SCRT, also involving ctDNA monitoring. IMPACT (NCT06032305) is a phase II trial (*N* = 40) investigating the addition of apatinib to SCRT + ICIs to evaluate immune infiltration and TME changes. STARS‐RC04 (NCT06342801) is a phase II trial (*N* = 50) of SCRT + CAPOX + sintilimab in patients with pMMR/MSS, focusing on cCR and NOM potential. TIMENT‐R (NCT05507112) is a phase II trial (*N* = 100) assessing LCRT with and without tislelizumab, exploring pCR and the effect of ICI timing during radiotherapy. PRIME‐RT (NCT04621370) is a phase II study (*N* = 48) incorporating durvalumab to SCRT or LCRT regimens for high‐risk LARC, assessing CR and immune modulation.

**TABLE 2:** Ongoing clinical trials integrating immune checkpoint inhibitors with total neoadjuvant therapy in proficient mismatch repair rectal cancer.

Trial name	NCT number	Phase	Registration year	Sample size	Study design	Treatment plan/intervention	Primary endpoint	Biomarker and translational research
*Phase III trials*								
STELLAR II	NCT05484024	III	2022	588	Randomized, parallel assignment	SCRT + CAPOX or FOLFOX ± sintilimab (PD‐1 inhibitor)	CR	Not specified in detail
PRECAM‐R	NCT05752136	III	2023	108	Randomized, parallel assignment	SCRT → CAPOX ± envafolimab (PD‐L1 inhibitor)	pCR	Not specified; DFS and OS follow‐up are secondary outcomes
UNICORN	NCT05922587	III	2023	280	Randomized, parallel assignment	SCRT → camrelizumab + CAPOX vs. conventional LCRT	DFS^a^	Not specified; secondary outcomes: OS, QoL, organ preservation
TORCH‐2	NCT06166016	III	2023	220	Randomized, parallel assignment	SCRT → toripalimab (PD‐1 inhibitor) + chemotherapy in varying sequences	pCR	PD‐L1 expression, tumor‐infiltrating lymphocytes, and other potential predictive factors
TNTi	NCT06229041	III	2023	472	Randomized, parallel Assignment	TNT ± camrelizumab (PD‐1 inhibitor)	pCR	Not specified; standard analyses for high‐risk features (MRF, EMVI, LN status) likely
*Phase II trials*								
Short‐course radiotherapy combined with CAPOX and toripalimab for LARC (“SECRAL”)	NCT04522613	II	2020	36	Single‐arm	SCRT + CAPOX + toripalimab (PD‐1 inhibitor)	pCR	Not specified; routine immunologic and correlative assessments are likely
PRIME‐RT	NCT04621370	II	2020	48	Randomized	High‐risk LARC (EMVI+ or lateral LN involvement)‐ SCRT or LCRT + durvalumab (PD‐L1 inhibitor)	pCR	Not specified; aims to improve local/distant control, possible immune correlates
Neoadjuvant toripalimab combined with CAPOX and radiotherapy for LARC	NCT04820953	II	2021	55	Single‐arm	Toripalimab (PD‐1 inhibitor) + CAPOX + radiotherapy (neoadjuvant)	pCR	Not specified; tumor/blood‐based biomarker sampling may be performed
Neoadjuvant radiochemotherapy ± durvalumab or SBRT ± durvalumab in T4b rectal cancer	NCT05057013	II	2021	80	Randomized	Arm A: chemoradiotherapy + durvalumab‐ Arm B: SBRT + durvalumab	pCR	Not specified; includes R0 resection rate, pCR, toxicity; immune correlates may be explored
TRIUMPHS (neoadjuvant tislelizumab, radiotherapy, and chemotherapy in LARC)	NCT05227982	II	2022	142	Randomized	Experimental: chemoradiotherapy + tislelizumab‐ Control: standard neoadjuvant chemoradiotherapy	pCR	Not fully detailed; may include immune phenotyping, tumor/blood biomarkers for response prediction
TIMENT‐R	NCT05507112	II	2022	100	Randomized	LCRT → tislelizumab (PD‐1 inhibitor)‐ compares delayed ICI administration vs. standard approach	pCR	Focus on immune activation post‐radiotherapy; details not fully disclosed
Neoadjuvant tislelizumab combined With radiotherapy in LARC	NCT05963831	II	2023	40	Single‐arm	SCRT + CAPOX + tislelizumab (PD‐1 inhibitor)	pCR	Planned correlative studies (e.g., PD‐L1 status, immune cell infiltration)
IMPACT	NCT06032305	II	2023	40	Single‐arm	SCRT + ICI + anti‐VEGF (apatinib)	pCR	Immune microenvironment assessment (cell infiltration), toxicity profile
NECTAR‐2	NCT06235599	II	2023	50	Single‐arm	Tislelizumab (PD‐1 inhibitor) + TNT‐ includes an arm combining SCRT + ICI + chemotherapy	pCR or cCR (protocol‐dependent)	Circulating tumor DNA (ctDNA) measurement for response prediction
STARS‐RC04	NCT06342801	II	2023	50	Single‐arm	SCRT + CAPOX + sintilimab (PD‐1 inhibitor)	cCR	Not specified; focuses on long‐term outcomes (bowel function, QoL)

*Note:* Ongoing clinical trials are investigating the combination of immune checkpoint inhibitors (ICIs) and total neoadjuvant therapy (TNT) for proficient mismatch repair (pMMR)/microsatellite stable (MSS) rectal cancer. The table includes key trial characteristics such as study design, treatment protocols, primary endpoints, and biomarker research. This table lists representative TNT‐ICI studies identified through a search of *ClinicalTrials.gov* conducted on 20 April 2025. This table lists representative studies identified through a search of *ClinicalTrials.gov* conducted on 20 April 2025.

Abbreviations: CAPOX, capecitabine + oxaliplatin; cCR, clinical complete response; CR, complete response; DFS, disease‐free survival; EMVI, extramural venous invasion positive; EMVI+, extramural venous invasion positive; ICI, immune checkpoint inhibitor (PD‐1, PD‐L1, etc.); LARC, locally advanced rectal cancer; LCRT, long‐course chemoradiotherapy; LCRT, long‐course radiotherapy; LLND, lateral lymph node dissection indicated; LN, lymph node; MRF, mesorectal fascia; OS, overall survival; pCR, pathological complete response; QoL, quality of life; SCRT, short‐course radiotherapy; TNT, total neoadjuvant therapy; VEGF, vascular endothelial growth factor.

^a^ UNICORN's primary endpoint is often cited as DFS, with OS, QoL, and organ preservation rates as secondary endpoints.

Collectively, these trials will provide critical insights into the optimal use of ICIs for pMMR/MSS LARC, exhibiting the potential to shape future treatment recommendations and redefine the standard of care by improving response rates and enabling organ preservation and personalized therapy based on predictive biomarkers.

## 5 Conclusions

TNT combined with ICIs is emerging as a promising treatment strategy for pMMR/MSS rectal cancer. Recent phase II and phase III trials have demonstrated meaningful short‐term improvements in both pCR and cCR rates, together with an expanded potential for organ preservation. Mechanistically, SCRT‐based or CRT‐based TNT converts an immunologically “cold” tumor microenvironment into a “hot” one: radiotherapy induces immunogenic cell death, enhances antigen presentation, increases tumour‐infiltrating lymphocytes, and up‐regulates PD‐L1, thereby augmenting the activity of PD‐1/PD‐L1 blockade. These effects appear to be amplified by lymphocyte‐sparing radiation schedules and by de‐escalation of systemic chemotherapy. Ongoing trials are now refining the optimal sequencing of radiotherapy, fractionation scheme, and chemotherapy intensity required to maximize efficacy while minimizing toxicity. Until mature survival data are available from the current phase III studies, however, the routine use of TNT combined with ICIs should be regarded as investigational.

## Author Contributions

**Yoshinori Kagawa:** conceptualization, writing – original draft, writing – review and editing. **Jun Watanabe:** writing – review and editing, conceptualization. **Koji Ando:** writing – review and editing, conceptualization. **Caleah Kitchens:** writing – review and editing, conceptualization. **Aron Bercz:** writing – review and editing, conceptualization, writing – original draft. **J. Joshua Smith:** writing – review and editing, conceptualization.

## Ethics Statement

The authors are accountable for all aspects of the work and ensure that questions related to the accuracy or integrity of any part of the work are appropriately investigated and resolved.

## Conflicts of Interest

Yoshinori Kagawa declares conflicts of interest with Lilly, Sanofi, Takeda, Merck, Taiho, MSD, Chugai, Bayer, and Ono. Jun Watanabe declares conflicts of interest with Johnson & Johnson, Medtronic, Eli Lilly, Takeda Pharmaceuticals, TERUMO, and Stryker Japan. J. Joshua Smith declares conflicts of interest with Intuitive Surgical, Guardant Health and Foundation Medicine, Johnson & Johnson, Urogen, Regeneron, and GlaskoSmithKline. Koji Ando, Caleah Kitchens, and Aron Bercz do not have any conflicts of interest. Jun Watanabe is an editorial member of the Annals of Gastroenterological Surgery.
